# Comprehensive energy demand and usage data for building automation

**DOI:** 10.1038/s41597-024-03292-2

**Published:** 2024-05-08

**Authors:** Philipp Heer, Curdin Derungs, Benjamin Huber, Felix Bünning, Reto Fricker, Sascha Stoller, Björn Niesen

**Affiliations:** 1 Swiss Federal Laboratories for Materials Science and Technology, Urban Energy System Laboratories, UESL, Dübendorf, Switzerland; 2 Swiss Federal Laboratories for Materials Science and Technology, Scientific Management Support, Dübendorf, Switzerland

**Keywords:** Energy modelling, Energy and behaviour

## Abstract

Buildings are essential in satisfying our daily need for comfort (privacy, protection from weather, etc.) and are responsible for almost half of the world’s total energy consumption. Research at the interface of room comfort and energy efficiency is of critical societal importance. At the same time, there is a lack of publicly available data to optimize important building functions automatically. It is only through data-driven approaches that building automation becomes financially affordable and achieves widespread adoption. In this publication, measurement data from three buildings of the NEST platform are made publicly available. The dataset includes detailed information on energy consumption (electricity, heating, cooling, domestic hot water), building operation (set points, valve openings, windows), and occupant practice (e.g., presence, operation of blinds and kitchen, showering patterns). All data have been measured over four years and with a temporal resolution of 1 minute. This combination of information allows learning the function of different building types (office and residential) and thus addresses important research gaps.

## Background & Summary

Buildings play a fundamental role in the lives of most. On average, we spend 90% of our time in buildings^1^, which are in turn responsible for approximately 40% of the world’s total energy consumption^2^. Smart building automation, therefore, bears the potential for saving considerable amounts of energy^3^ and for increasing our well-being, for instance, by optimizing lighting^4^, controlling room climates^5^, or increasing security^6^. Despite its vast potential, however, digitalization has only marginally entered the building sector yet^7^. One important reason is the limited availability of representative information to develop building automation.

In recent years, a large number of building-related data sets have been published under an open-access policy. Examples are detailed measurements of electric power, for instance, to train models for non-intrusive load monitoring^8,9^, different types of energy demands from relevant systems of individual buildings^10,11^, or information on occupant behavior in buildings^12–14^. However, to date, few openly available datasets exist that combine three different aspects, namely i) detailed information on the consumption of all relevant energies in buildings (electricity, heat, and domestic hot water), ii) detailed monitoring of factors that allow an analysis of user practice, as well as iii) information on the operation of buildings. This combination of information fosters the development of automation concepts that can learn and control key functionalities of buildings. In this publication, we aim to close this gap with data collected at the NEST (Next Evolution in Sustainable Building Technologies) research platform.

The dataset published in this article comprises three buildings of different use types. An office building, a building that contains offices and meeting rooms, and one residential building. The measurement data covers four whole years, with a temporal resolution of one minute. Measurements for all relevant energy flows are made available, as well as information on different types of usage patterns (e.g., occupancy, window openings, blind control, cooking, showering, ambient air temperature). Finally, we allow access to the set points of the HVAC system (heating, cooling, and ventilation). This information allows to comprehend how the system has been operated.

Research in building automation is currently experiencing a shift from physical building models and rule-based approaches towards more data-driven procedures^15^. These novel procedures allow for learning complex building behavior from historical measurement data. First results from our research at NEST show that rule-based approaches for HVAC control can be outperformed - with regard to energy efficiency and room comfort - at the same time, the effort of creating physical building models can be omitted^16^.

However, for a successful practical application of such approaches, much research is needed to foster the robustness of data-driven building automation. Examples are testing of different statistical approaches (machine learning, deep learning, reinforcement learning, transfer learning), comparing various buildings and building use types, incorporating user behavior and cost-benefit evaluations, to name a few. We believe that the dataset presented in this study will allow researchers to progress in said areas and thus pave the way for sustainable digitalization in the building sector.

Describing the entire NEST dataset is beyond the scope of this publication. Additional, specific measurement points for the three buildings described here can be provided on request, as well as comprehensive measurements for three additional buildings. All measurement data from the NEST can be accessed and used for research purposes.

## Methods

All measurement data published in this publication stems from the NEST research platform. We will briefly introduce NEST, with a particular focus on the selected buildings and the measurement infrastructure.

### NEST building

The NEST^17^ research platform of Empa (Swiss Federal Laboratories for Material Sciences and Technology) and Eawag (Swiss Federal Institute of Aquatic Sciences and Technology), both branches of the ETH Domain (Swiss Federal Institute of Technology), is a living lab environment for testing new technologies and materials under real conditions. NEST has a modular structure. On a multilevel framework, the so-called Backbone, which has space for up to 12 Units. Each Unit can be considered an independent building, as they draw or produce energy without taking the other buildings into account, possess their own building automation solutions, and are thermally insulated between each other and the Backbone. These buildings are connected to common, district-size generation plants for heating, cooling, and ventilation.

The NEST buildings have different usage types. Currently, NEST counts three residential buildings, one building for spa and fitness, and several buildings with offices and meeting rooms. Each building is dedicated to a specific topic, such as circular economy, wood construction, solar energy, or digital construction processes. All NEST buildings are actively used by occupants. In total, around 10 people work at NEST, another 10 use it as a place to live, approximately 1'000 people attend meetings each year, and some 10'000 people visit NEST each year on guided tours. An important functionality of NEST is dedicated to the collection and analysis of building, energy, and usage data. The ehub platform was developed for this purpose.

### ehub platform

**Fig. 1:**
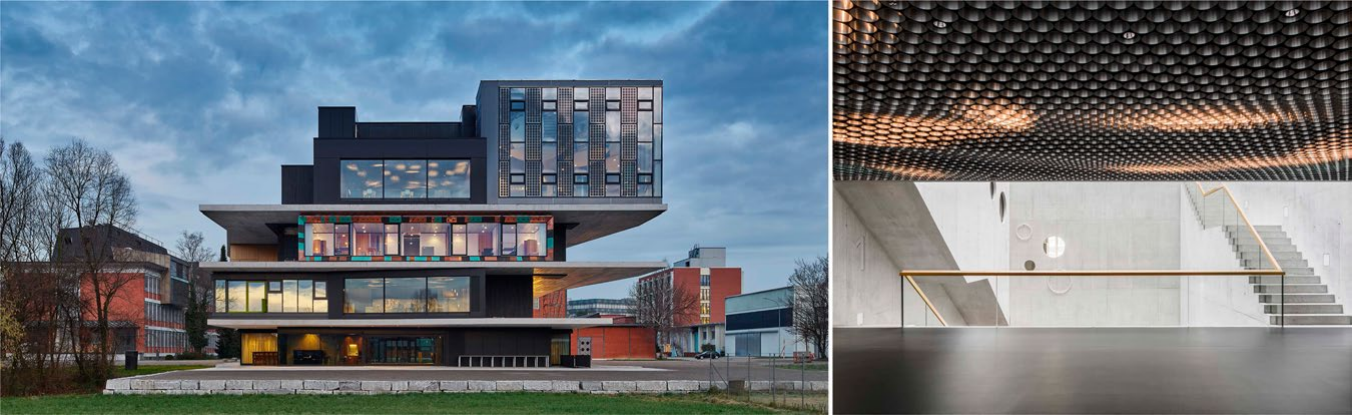
The NEST platform. Left: NEST building as seen from the East. The colorful framed building on the second floor is UMAR (Source: Zooey Braun). Right: Interior shot of the NEST Backbone (Source: Roman Keller).

Ehub^18^ (Energy Hub) is a technology platform for researching energy-related topics on the scale of buildings, districts, and cities. The central component of ehub is the measurement infrastructure for collecting, storing, and processing all relevant energy flows (e.g., electricity, water, air) and operating states (e.g., room temperatures, position of blinds, opening of valves, etc.) at NEST and other platforms. In its current state, ehub accesses 10'000+ data points with a sample rate of one minute. The data can be retrieved either in real-time, via a REST API, or as histories from an SQL database (Structured Query Language). Interested researchers can request for an ehub user key^19^ and then access NEST’s measurement data, as well as a rich repository with metadata (e.g., dashboards, 3D models, technical schematics, etc.). In addition, NEST can be remote controlled with own algorithms. Thereby new approaches, for instance, for the energy-efficient operation of buildings can be tested in practice Fig. 1.

### NEST Buildings

**Fig. 2:**
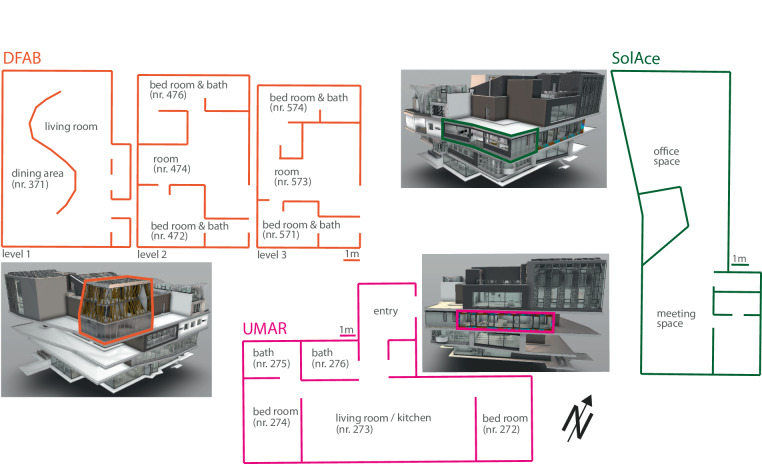
Selected NEST Buildings. 3D views and floor plans of the three NEST buildings DFAB, SolAce, and UMAR, contained in this open data publication. The room numbers and labels are used to reference the measurement points in the data set.

The measurement data in this publication stems from three different NEST buildings: DFAB, SolAce, and UMAR^19^. The selection represents a variety of usage types, technological prerequisites, and possibilities for regulatory measures. The floor plan of the three buildings is sketched in Fig. 2. Detailed floor plans, as well as 3D models of the units, can be downloaded from the associated repository^20^.

The selection of these three buildings allows researchers to, for instance, develop a data-driven building automation with the data of one building and test its robustness with the other two buildings. Additionally, the three buildings allow to observe and incorporate different dimensions of user practice in building automation, which is a topic frequently discussed in research^21^. Table 1 summarizes the characteristics of the three buildings.

**Table 1:** Characteristics of each of the three buildings.

Usage type	UMAR	DFAB	SolAce
	residential	residential	meeting room / office
Rooms (Bold font shows the rooms which are included in this publication)	Entry, **living room** / **kitchen (nr. 273),** **two bedrooms (nr. 272, 274)** and two bathrooms.	Living room, **dining area (nr. 371),** **two lounges (nr. 474, 573),** **four bedrooms (nr. 472, 476, 571, 574)**, each with a private bath.	**Office space,** **meeting space**.
Total area	155m^2^	240m^2^	103m^2^
Date of construction	02.2018	02.2019	09.2018
Building technology	Ceiling heating/cooling, high temperature domestic water storage tank.	Photovoltaic panel, heat pump boiler, floor heating/cooling, automatic ventilation, heat recovery from hot water.	Photovoltaic panel, solar thermal panel, ceiling heating/cooling, automatic ventilation, window shading, domestic hot water and storage.
Occupant related information	Window openings, temperature set point for heating/cooling, domestic hot water consumption, total electricity consumption.	Temperature set point for heating/cooling, total electricity consumption and electricity consumption of the kitchen, water consumption showers.	Movement sensor for presence detection, Position of blinds, temperature set point for heating/cooling.
Construction	Roof and floor construction with a wood beam layer insulated with rock wool and a vapor barrier. Ventilated façade with a wood beam layer insulated with rock wool and a vapor barrier. The window front is triple glazed.	Three-story house with flat roof. The 1st floor wood beam construction is insulated with rock wool and two triple glazed glass facades. The 2nd and 3rd floor wood beam structure is insulated with aerogel.	Roof and floor construction with a wood beam layer insulated with rock wool and a vapor barrier. Ventilated façade with a wood beam layer insulated with rock wool and a vapor barrier. The window front is triple glazed.

Room numbers in brackets foster comparisons with the sketch in Fig. 2. More detailed information on the sensor types and the exact measurements is contained in Table 2.

Occupancy levels of the two residential units UMAR and DFAB can be retrieved from can be downloaded from the associated repository^22^.

## Data Records

The measurement data are made available can be downloaded from the associated repository^23^.

The repository contains measurement information for the three NEST buildings for the measurement period of four years (01 July 2019 to 30 June 2023) and a temporal resolution of one minute. Each building is represented by a series of measurement points. The measurement data for each full year and for each building is stored in an individual CSV file. The data have table wide format. The first column contains the time tag. The formatting of the time tag is *Year-Month-Day Hour:Minute:Second*, for example *2019-07-01 01:16:00*. The time is recorded in local time, i.e., European Central Time (UTC + 1 in winter and +2 in summer, respectively). The measurement data for each measurement point are stored in individual columns. The header of each column corresponds with the UID in Table 2. Additionally, one metadata file is available for each building that contains more information for each measurement point.

**Table 2:** Metadata information for each measurement point included in the published data set including the first and last entry.

UID	Building	Type	Details	Sensor	Unit	Resolution	Calibration
temp_amb	Backbone Roof	Air temp.	Ambient air temperature, Type: PT1000 Range: −30… + 60 °C Accuracy: + −1 °C @ wind speed 2 m/s and between −5… + 25 °C measured on NEST’s rooftop.	Thies Clima WSC 11	°C	0.15	No
…	…	…	…	…	…	…	…
shower_471, shower_571	DFAB	Hot water	Hot water consumption of two showers.	Ifm SM6100	l/min	0.1	NA

In the following, additional information for each measurement point is summarized, all pre-processing steps required for further application of the data are discussed, and, finally, the data is subject to a quality assessment.

### Measurement points

Table 2 shows the metadata information of all measurement points made accessible in this publication. The full table itself can be found in the provided repository^24^.

Only power sensors have been calibrated. These sensors follow the Measurements Instrument Directive (MID) guidelines^25^.

### Pre-processing

Very little processing was applied to the raw data from NEST. Most importantly, a physically meaningful range of values was defined for each type of measurement point. Values outside this range are set to missing value (i.e., *NA*). The aim of this pre-processing is to distinguish physically possible from impossible measurements. Table 3 shows the metadata information of minimum and maximum values that constitute the rages per type of measurement point. The full table itself can be found in the provided repository^24^.

**Table 3:** Metadata information of minimum and maximum threshold values for each type of measurement point used in the pre-processing set including the first and last entry.

Type	Description	Min	Max	Unit
Valve position	All valves of the floor heating are either open (=100%) or closed (=0%).	0	100	%
…	…	…	…	…
PV Production	A total of 17m^2^ of façade PV panels produce electricity for SolAce with 2.2 kWp. Negative values mean electricity production (i.e., feed-in), small positive values mean consumed energy by the PV (due to e.g., stand by losses during night time).	−10	1	kW

Values outside the threshold values are set to missing values (NA).

#### Domestic hot water

The absolute consumption of domestic hot water at UMAR is measured in cubic meters since the installation of the meter. To obtain the consumption per minute, the previous reading of the meter must be subtracted from the current reading. This is often referred to as “differencing” of a measurement series. The shower consumption at DFAB is an event-based measure. During a shower event, the temporal resolution of the measurement point is increased to 1 second. To obtain minute values and thus simplify comparability with all other measurements, all measurements within one minute are summed.

Further processing steps, such as interpolation of missing measurement values or a probabilistic outlier detection, were deliberately omitted. Respective processing of the data is often domain specific and is therefore left to the user of the data.

### Quality assessment

**Fig. 3:**
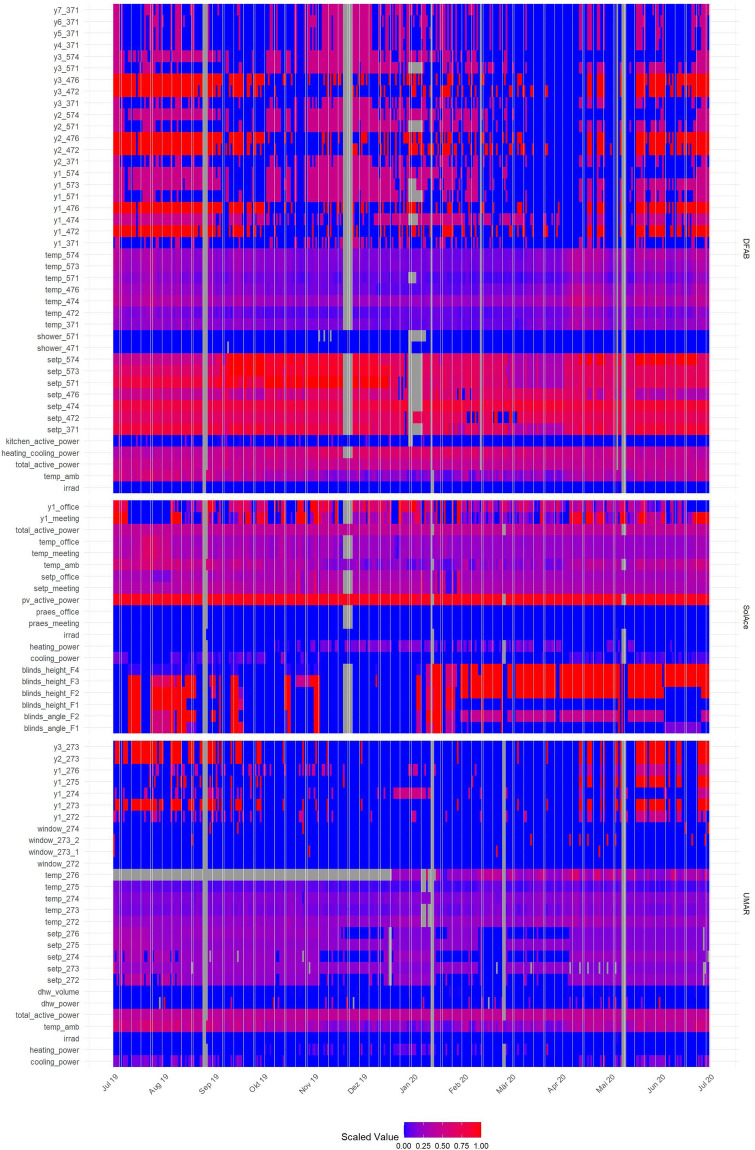
Data overview from July 2019-June 2020. All measurements from all measurement points. The values from each measurement point are scaled to a range between 0 and 1. The grey color represents missing values.

Figure 3 shows all measurements from all measurement points with a sampling rate of one minute, during the first year (approximately 47 M measurements). For better comparability, the measurements from each measurement point are scaled, such that the minimum value is 0 and the maximum value is 1. Grey color highlights missing values.

In total, some 3.5% of all measurements are missing (grey color) over all years. In the first year seen in Fig. 3 shows that missing values are not randomly distributed over the observation period. Several time periods can be identified where many measurement points simultaneously show missing values (vertical, grey bars). Between August and September 2019, for example, the cloud provider migrated the system, which lead to a maintenance window of two days. In December 2019, the connection between the OPC UA and the SQL database was interrupted and the memory of the buffer system exceeded its limit.

Sometimes only individual sensors fail. One example is the temperature in Room 276 at UMAR (*temp_276*). Here it was only noticed after several months that the sensor has no battery and therefore does not transmit any values. Such problems have recently been intercepted by standardized pre-processing and an alarm system.

The color patterns for the different measuring points in Fig. 3 correspond to what one would expect from the individual sensor types. Temperature measurements, for example, are continuously distributed over the range of values (e.g., *temp_274*), while the windows are mostly closed (e.g., *window_272*), the kitchen is often not in use (*kitchen_active_power*), and hot water is only occasionally consumed (e.g., *dhw_volume*). The corresponding measuring points, therefore mostly show the value 0 (blue).

Similar observations can be made for the other three years provided. The quality assessment for all years can be downloaded from the associated repository^26^.

## Technical Validation

The technical validation is structured into two parts. First, the relation between ambient air temperature, heating and cooling energy input, and the resulting room temperature is validated. This is an important aspect of the data since it for instance allows implementing novel building automation for heating and cooling, which is the largest energy consumption in buildings (i.e. 75% in Switzerland^27^). In a second step, information relevant for studying user patterns is explored. User patterns are often considered to have major impact on energy efficiency of buildings^21^. The technical validation is shown for the first of the four provided years of data. The technical validation for all years can be downloaded from the associated repository^26^.

### Heating, Cooling and Temperatures

**Fig. 4:**
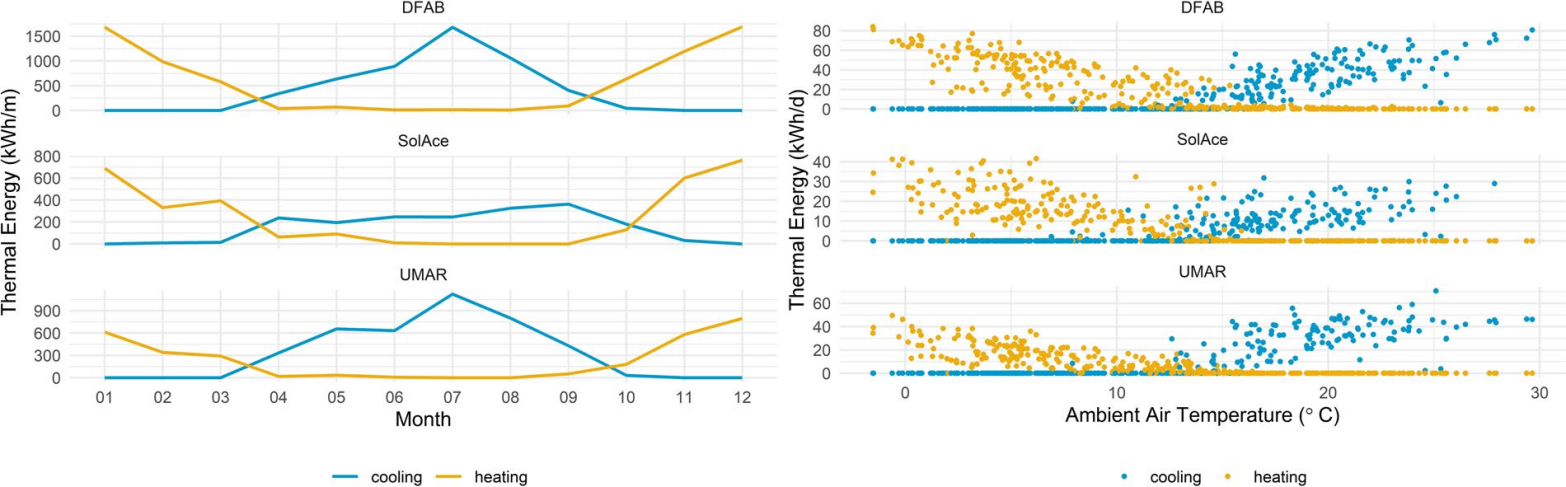
Heating, cooling and ambient air temperature. Left: Seasonal patterns in the consumption of heating and cooling energy in the three buildings. Right: Relation between ambient air temperature and energy consumption for heating and cooling.

The demand of heating and cooling energy is dependent on the ambient air temperature. Figure 4 shows the seasonal variation in heating and cooling demand (left, per month), as well as the direct relation between ambient air temperature and cooling and heating demand for daily aggregates (right).

On the one hand, a pronounced annual pattern can be observed. Cooling demand is peaking in summer, while heating demand reaches its maximum in winter. In UMAR, the maximum cooling demand exceeds the demand for heating energy. For Switzerland, this is a notable pattern and is explained in more detail later in the text. The comparison between energy demand and ambient air temperature, on the other hand, shows that ambient air temperature only explains part of the variation in energy consumption. For a given ambient air temperature, considerably different cooling and heating demands can result. The total consumption of heating and cooling energy per building, year, and square meter is summarized in Table 4.

**Table 4:** Heating and cooling Energy per year and square meter in the three buildings at NEST.

	Heating Energy/Cooling Energy [kWh/ym2]			
	July 2019-June 2020	July 2020-June 2021	July 2021-June 2022	July 2022-June 2023
DFAB	29/21	30/21	26/23	24/22
SolAce	30/18	46/20	29/25	25/27
UMAR	19/26	33/29	30/21	30/26

The heating energy consumption of all three buildings is comparable to the threshold values of European energy efficiency standards. The Minergie-P^28^ standard in Switzerland, for instance, requires heating demand to be smaller than 35 kWh/ym^2^. Cooling demand is usually expected to be around 30 to 50% of heating demand in the present climatic zone. All three buildings thus have a considerably high cooling demand. In UMAR, cooling demand even exceeds heating demand. The reason for this effect is a large window area, causing solar radiation to have a great impact on the indoor climate and thus increasing cooling demand in summer and reducing heating demand in winter. This behavior can be considered typical for newer building types^29^.

**Fig. 5:**
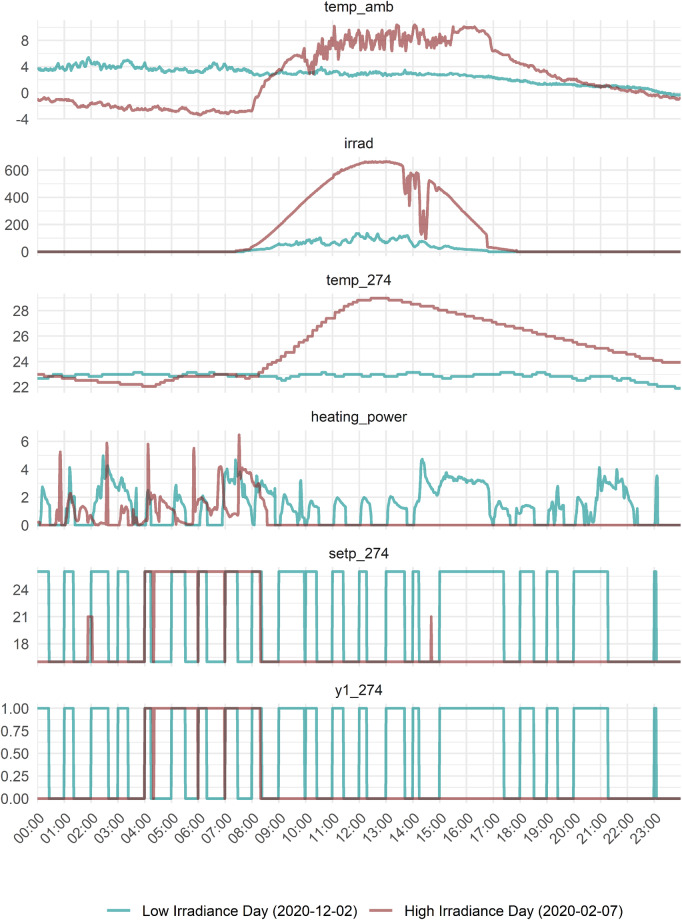
The impact of solar radiation. Comparison of all temperature-relevant measurement points at UMAR for two winter days, one with low and one with high solar irradiance, respectively. Temp_amb, temp_274 and setp_274 in C°, irrad in W/m^2^, heating_power in kW, y1_274 in close/open.

The more complex relation between heating demand, ambient air temperature, and solar radiation is best illustrated with an example. In Fig. 5, the heating demand of UMAR for two winter days is compared. The two days are characterized by quite similar outdoor air temperatures. However, one day is cloudy (blue line), and the other day the sky is clear, and solar irradiance is high (red line).

The day with high solar irradiance shows that the increase in solar irradiance (*irrad*) at around 8am immediately causes the room temperature to rise (*temp_274*). On a sunny winter day, room temperatures can reach some 30 °C. Despite relatively low ambient air temperatures (around −2 °C, *temp_amb*), no additional heating power is required (*heating_power*) after sunrise on a clear winter day. In contrast, on cloudy winter days with low solar irradiance, the building requires a varying heating energy provision over the day.

From a more technical point of view, Fig. 5 shows that changes of the set point of the heating system (*setp_274*), which are automatically adjusted in this example, have an immediate impact on the opening of the valves of the ceiling heating (*y1_274*), which in turn leads to an increased input of heating power (*heating_power*).

The patterns described in this chapter cover different temporal granularities, from annual to hourly, and show that the measured data are consistent with the physical characteristics of the buildings.

### User patterns

The data set contains a series of measurements that allow the study of user patterns and to, for instance, account for it in building automation. In the following, examples of user related information are shown for all three buildings.

#### UMAR

**Fig. 6:**
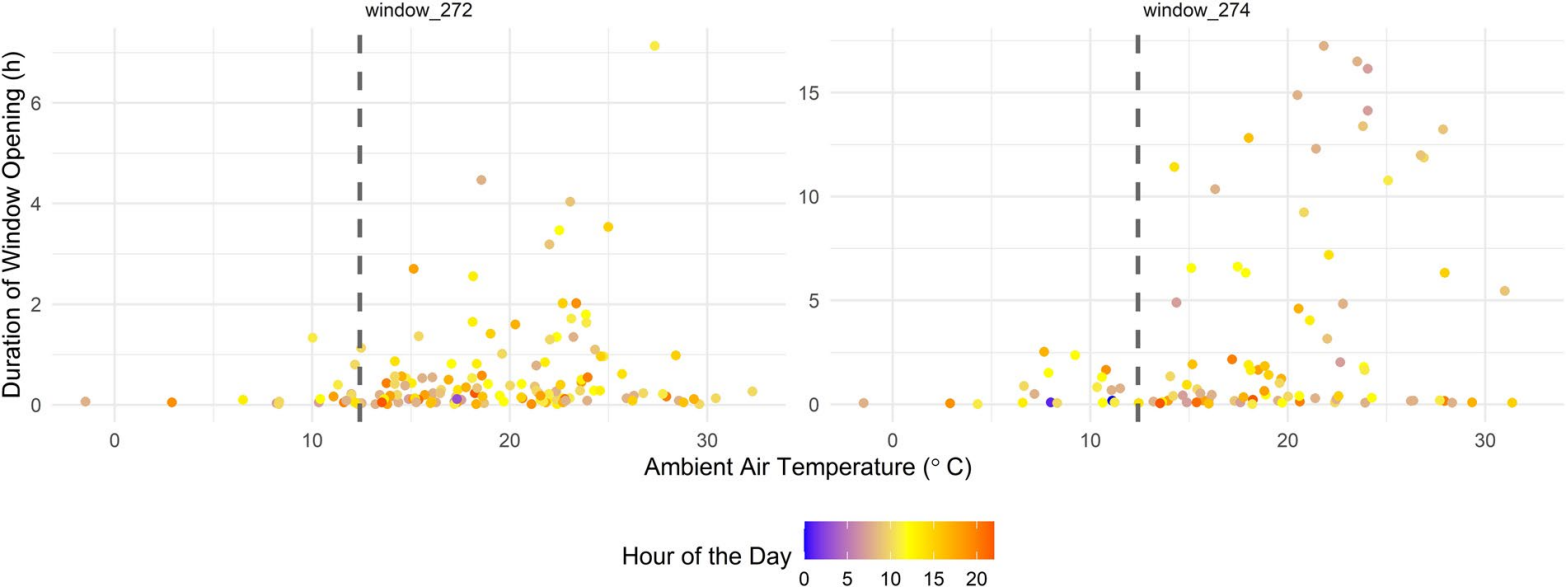
UMAR windows openings. The duration of each window-opening event at UMAR is compared to the average ambient air temperature. The color represents the hour of the day, with yellow colors being associated with midday. The dashed vertical line shows the average ambient air temperature throughout the year. Room 272 counts 145 openings, while in Room 274 the window has been opened some 99 times.

In Fig. 6, all events of window openings in the UMAR building (*window_272* and *window_274*) are represented, with respect to the duration of the opening and the current ambient air temperature for the first year of data. Room 272 counts 145 openings, while in Room 274 the window has been opened 99 times during the observation period of the first year. Both rooms were occupied during the whole year.

From Fig. 6, one can see that the majority of long window openings are associated with ambient air temperatures above a certain threshold temperature (i.e., to the right of the dashed line that represents the average ambient air temperature at NEST’s location). Long opening periods are more frequent in Room 274, as compared to Room 272. Windows are opened in the morning (brownish dots) and left open for most of the day. This is just one indication for the variation between different users of buildings.

#### SolAce

**Fig. 7:**
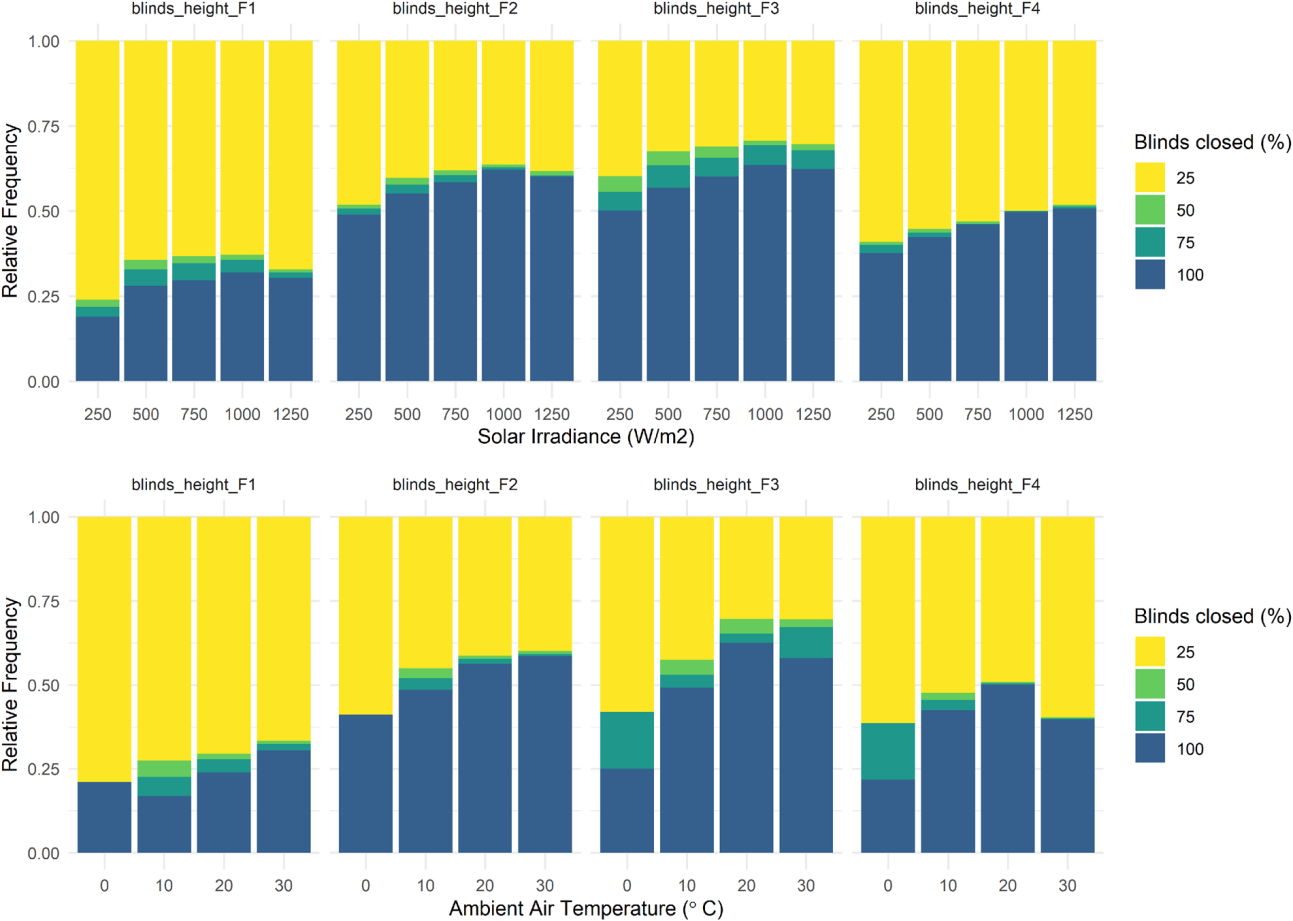
SolAce blinds operation. Blinds states per solar irradiation (first row of graphs) and ambient air temperature class (second row of graphs). Labels refer to the upper bounds of the temperature and irradiation bins, respectively. Light color indicates open blinds, while dark color represents closed blinds (upper bounds of blinds state bins are shown in the legend, e.g., 25 means 0–25% closed).

Figure 7 shows blinds states (colors) per solar irradiation (first row of graphs) and ambient air temperature (second row of graphs), for all four blinds at SolAce (four columns of graphs).

Blinds are either fully closed (dark blue) or open (yellow). Half-open blinds are rarely observed. There is a trend of blinds being mostly open if ambient air temperature and solar irradiance are low (i.e., winter). On summer days with high solar irradiance, blinds are mainly used to manually control the room temperature, as the incident sunlight causes a lot of heating in the room (c.f. Figure 5). Blinds are therefore often closed. There is no automatic operation of blinds to control room temperatures. The exposed window area is 24.4 square meters. Of this, 6.4 square meters are equipped with fabric blinds and 14 square meters with slat blinds.

**Fig. 8:**
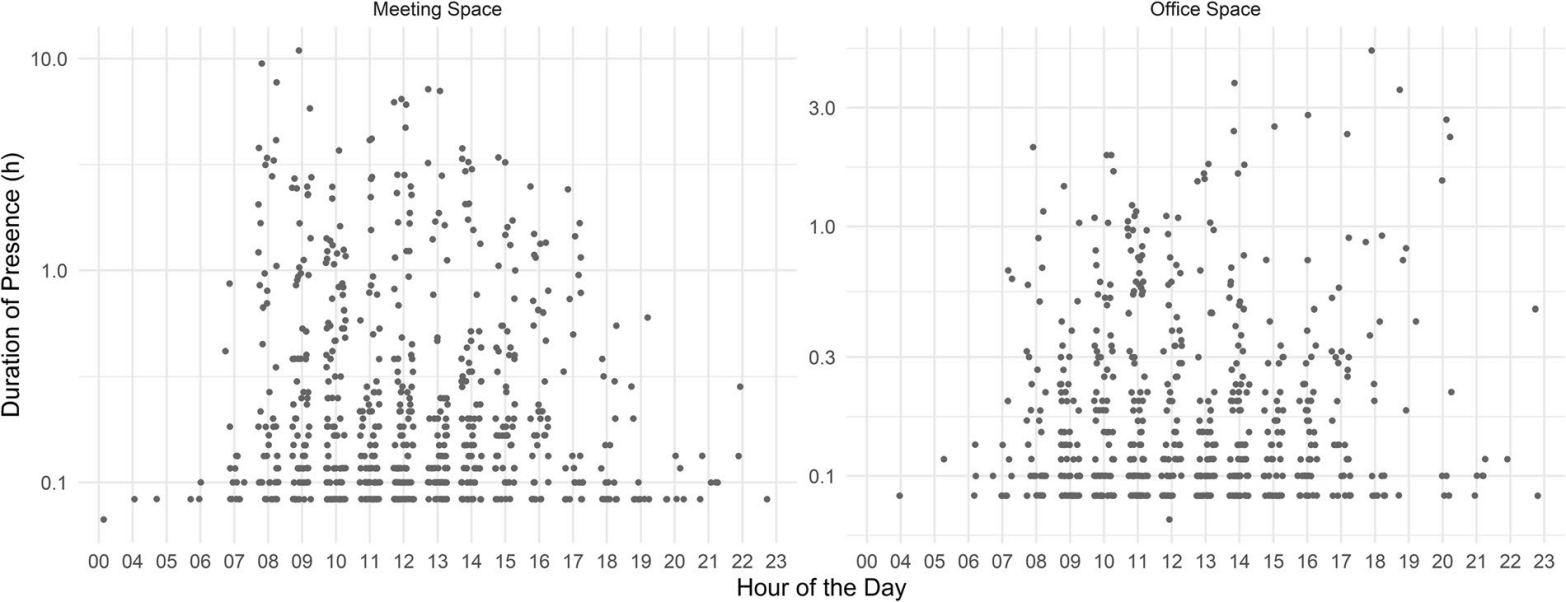
SolAce user presence. Each presence in SolAce’s meeting and office space (n = 1350) is represented using its duration (logarithmic) and the hour of the day, when the presence started.

Additionally to the monitoring of the blinds, SolAce is equipped with presence sensors. The presence detector is an infrared sensor which can detect moving persons, also sitting activities are detected. The building counts some 1'350 unique “presence” in the observation period from July 2019 to July 2020. These are time windows with at least one person present in one of the two rooms. Figure 8 visualizes each unique presence at SolAce, depending on the hour of the day (x-axis) and the duration of the presence (y-axis, logarithmic).

As expected, there are only few and short visits of the meeting and office space at night (e.g., security service of NEST). Most visits of SolAce are during office hours and have a duration of around 30 Minutes. The many short attendances can be explained by the tours that are offered at NEST. SolAce is often visited during these tours. There are then 5 to 20 people in the building for about 5 to 10 minutes.

#### DFAB

**Fig. 9:**
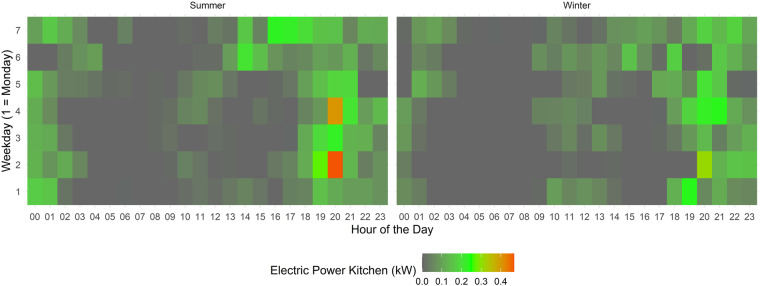
DFAB kitchen usage. Electric power usage of the kitchen at DFAB.

Figure 9 shows the average electric power usage of DFAB’s kitchen for the hours of the day (x-axis), the weekdays (y-axis), and summer and winter separately.

The highest power consumption for the kitchen usually occurs in the evenings, with smaller power peaks around lunchtime (Empa has a canteen, which is closed on weekends). Saturdays and Sundays (Weekdays 6 & 7) show slightly higher power consumption in the afternoons, as compared to the working days. The power consumption is similar in winter and summer.

**Fig. 10:**
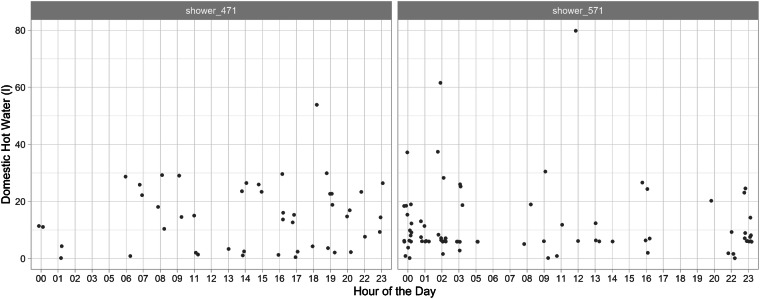
DFAB shower usage. Each shower event at DFAB is visualized against the hour of the day and the total domestic hot water consumption.

Figure 10 show all shower events in the two showers that are monitored in DFAB. The two showers each belong to a single-person apartment. An average shower event at DFAB uses 45 liters of domestic hot water. This is in agreement with findings from field studies in Switzerland^30^. Each shower event is plotted against the hour of the day (x-axis) and the total consumption of water (y-axis) for the two showers.

Occupants using Shower 471 (level 2 of DFAB) and 571 (level 3) show slightly different practices. Shower 571 is mainly used during the hours around midnight, while Shower 471 shows a more ordinary use pattern. It is mainly used during the evening hours. In total, each shower has been used around 200 times between July 2019 and July 2020.

## Usage Notes

Examples of computer code to read and process the data, and to reproduce the figures of this publication are referenced in the next chapter.

We have used the data from ehub, and in particular the publicly available data from this publication, for diverse approaches of data-driven building automation^16,31–35^. On the one hand, this supports the argument that ehub’s measurement data are well suited to fill an important data gap in building research. One pre-processed csv file per Unit (i.e., DFAB, Sprint, and UMAR) per year is provided.

In addition, the authors of the corresponding studies are happy to answer questions from interested researchers.

## Data Availability

R programming code used to create all visualizations in the above quality assessment and technical validation is available as R-Markdown files in the same repository as is used to share the measurement data^26^. One Rmd for the yearly Quality Assessment and one for each yearly Technical Validation is provided.

## References

[1] Klepeis NE (2001). The National Human Activity Pattern Survey (NHAPS): a resource for assessing exposure to environmental pollutants. J. Expo. Sci. Environ. Epidemiol..

[2] Mancini F, Lo Basso G, De Santoli L (2019). Energy use in residential buildings: Impact of building automation control systems on energy performance and flexibility. Energies.

[3] Minoli D, Sohraby K, Occhiogrosso B (2017). IoT considerations, requirements, and architectures for smart buildings—Energy optimization and next-generation building management systems. IEEE Internet Things J..

[4] Aghemo C, Blaso L, Pellegrino A (2014). Building automation and control systems: A case study to evaluate the energy and environmental performances of a lighting control system in offices. Autom. Constr..

[5] Aftab M, Chen C, Chau C-K, Rahwan T (2017). Automatic HVAC control with real-time occupancy recognition and simulation-guided model predictive control in low-cost embedded system. Energy Build..

[6] Granzer W, Praus F, Kastner W (2009). Security in building automation systems. IEEE Trans. Ind. Electron..

[7] Wong JKW, Ge J, He SX (2018). Digitisation in facilities management: A literature review and future research directions. Autom. Constr..

[8] Kriechbaumer T, Jacobsen H-A (2018). BLOND, a building-level office environment dataset of typical electrical appliances. Sci. Data.

[9] Medico R (2020). A voltage and current measurement dataset for plug load appliance identification in households. Sci. Data.

[10] Pipattanasomporn M (2020). CU-BEMS, smart building electricity consumption and indoor environmental sensor datasets. Sci. Data.

[11] Rashid H, Singh P, Singh A (2019). I-BLEND, a campus-scale commercial and residential buildings electrical energy dataset. Sci. Data.

[12] Paige F, Agee P, Jazizadeh F (2019). flEECe, an energy use and occupant behavior dataset for net-zero energy affordable senior residential buildings. Sci. Data.

[13] Mahdavi A, Berger C, Tahmasebi F, Schuss M (2019). Monitored data on occupants’ presence and actions in an office building. Sci. Data.

[14] Huebner GM, Mahdavi A (2019). A structured open data collection on occupant behaviour in buildings. Sci. Data.

[15] Amasyali K, El-Gohary NM (2018). A review of data-driven building energy consumption prediction studies. Renew. Sustain. Energy Rev..

[16] Bünning F, Huber B, Heer P, Aboudonia A, Lygeros J (2020). Experimental demonstration of data predictive control for energy optimization and thermal comfort in buildings. Energy Build..

[17] Empa. NEST - Homepage. *NEST – Gemeinsam an der Zukunft bauen* https://www.empa.ch/web/nest/ (2024).

[18] Empa. ehub - Homepage. *ehub – Energy Hub* https://www.empa.ch/web/energy-hub/ (2024).

[19] Fricker, R., Stoller, S. & Heer, P. Links to nestcloud access form and Solace, UMAR and DFAB wiki pages, 10.6084/M9.FIGSHARE.C.7191198. (2024).

[20] Heer, P., Stoller, S. & Fricker, R. NEST - DFAB, UMAR, SolAce Floor Plans and Sensor Placements. 10.6084/M9.FIGSHARE.24551983 (2023).

[21] Agarwal, Y. *et al*. Occupancy-driven energy management for smart building automation. in *Proceedings of the 2nd ACM workshop on embedded sensing systems for energy-efficiency in building* 1–6 (2010).

[22] Stoller S, Heer P, Fricker R (2023). Figshare.

[23] Heer, P., Stoller, S. & Fricker, R. NEST Open Building Data for Energy Demand and User Practice, 10.6084/M9.FIGSHARE.C.7178787 (2024).

[24] Heer P, Stoller S, Fricker R (2024). Figshare.

[25] European Commission. Measuring instruments (MID) - European Commission. *Internal Market, Industry, Entrepreneurship and SMEs* https://single-market-economy.ec.europa.eu/single-market/european-standards/harmonised-standards/measuring-instruments-mid_en (2024).

[26] Heer P, Derungs C (2023). Figshare.

[27] Kemmler, A. & Spillmann, T. *Analyse Des Schweizerischen Energieverbrauchs 2000–2019*. https://www.bfe.admin.ch/bfe/de/home/versorgung/statistik-und-geodaten/energiestatistiken/energieverbrauch-nach-verwendungszweck.html (2020).

[28] Minergie. Minergie-P - Minergie. *Zertifizierung im Minergie-P-Standard* https://www.minergie.ch/de/zertifizieren/minergie-p/ (2024).

[29] Grynning S, Gustavsen A, Time B, Jelle BP (2013). Windows in the buildings of tomorrow: Energy losers or energy gainers?. Energy Build..

[30] Tiefenbeck, V. *et al*. Steigerung der Energieeffizienz durch Verbrauchsfeedback: Abschlussbericht der ewz-Amphiro-Studie. (2013).

[31] Svetozarevic B (2022). Data-driven control of room temperature and bidirectional EV charging using deep reinforcement learning: Simulations and experiments. Appl. Energy.

[32] Bünning, F., Huber, B., Heer, P., Smith, R. & Lygeros, J. Improved day ahead heating demand forecasting by online correction methods. *Energy Build*. 109821 (2020).

[33] Bünning, F., Warrington, J., Heer, P., Smith, R. S. & Lygeros, J. Frequency regulation with heat pumps using robust MPC with affine policies. in *1st Virtual IFAC World Congress (IFAC-V 2020)* (2020).

[34] Khosravi, M., Schmid, N., Eichler, A., Heer, P. & Smith, R. S. Machine learning-based modeling and controller tuning of a heat pump. in *Journal of Physics: Conference Series* vol. 1343 12065 (2019).

[35] Gasser J, Cai H, Karagiannopoulos S, Heer P, Hug G (2021). Predictive energy management of residential buildings while self-reporting flexibility envelope. Appl. Energy.

